# Старт терапии этелкальцетидом у пациентов с ХБП5Д и вторичным гиперпаратиреозом: оценка предикторов эффективности в когортном ретроспективном исследовании

**DOI:** 10.14341/probl13563

**Published:** 2026-01-18

**Authors:** Е. В. Паршина, Р. П. Герасимчук, А. Ю. Земченков, А. Б. Зулькарнаев

**Affiliations:** Санкт-Петербургский государственный университет, Клиника высоких медицинских технологий им. Н.И. ПироговаРоссия; Saint Petersburg University, N.I. Pirogov Clinic of High Medical TechnologiesRussian Federation; Северо-Западный государственный медицинский университет им. И.И. Мечникова; Городская Мариинская больницаРоссия; Northwestern State Medical University n.a. I.I.Mechnikov; City Mariinsky HospitalRussian Federation; Городская Мариинская больницаРоссия; City Mariinsky HospitalRussian Federation; Московский областной научно-исследовательский клинический институт им. М.Ф. ВладимирскогоРоссия; Moscow Regional Scientific Research Clinical Institute n.a. M.F. VladimirskyRussian Federation

**Keywords:** вторичный гиперпаратиреоз, цинакальцет, этелкальцетид, аденома паращитовидной железы, hyperparathyroidism, secondary, cinacalcet, etelcalcetide, parathyroid adenoma

## Abstract

**ОБОСНОВАНИЕ:**

ОБОСНОВАНИЕ. Распространенность вторичного гиперпаратиреоза (ВГПТ) зависит от тактики его контроля и целевого уровня ПТГ. Для строго обоснованного выбора целей и тактики коррекции ВГПТ данных недостаточно.

**ЦЕЛЬ:**

ЦЕЛЬ. Ретроспективное когортное многоцентровое исследование проведено для оценки результатов применения этелкальцетида в условиях реальной клинической практики. Гипотезой стало предположение, что этелкальцетид будет через полгода эффективно снижать ПТГ, а исходные характеристики МКН-ХБП являются предикторами достижения целевого диапазона.

**МАТЕРИАЛЫ И МЕТОДЫ:**

МАТЕРИАЛЫ И МЕТОДЫ. В ретроспективное когортное исследование включены 302 пациента, получавшие этелкальцетид в 20 отделениях диализа Санкт-Петербурга.

Первичная конечная точка — доля пациентов, достигших целевого диапазона через 6 мес (300–599 пг/мл); вторичная — абсолютное и относительное снижение ПТГ. Сравнивали результаты в подгруппах с исходным ПТГ (<600, 600–1000, >1000 пг/мл) и с признаками автономии околощитовидных желез (ОЩЖ) и без них.

**РЕЗУЛЬТАТЫ:**

РЕЗУЛЬТАТЫ. Среди включенных пациентов в возрасте 56 (12) лет (мужчин 61%) при медиане срока диализа 36 (Q1–Q3 23–55) месяцев при исходном ПТГ 729 (548–957) пг/мл и при исходной кальциемии и фосфатемии 2,35 (0,25) и 1,97 (0,47) ммоль/л соответственно доля достигших целевого диапазона ПТГ при наличии признаков автономии ОЩЖ составила 48,0% vs. 85,6% в отсутствие автономии (p<0,001) в подгруппе с исходным ПТГ 600–1000 пг/мл. Соответствующее соотношение для пациентов с ПТГ выше 1000 пг/мл составляет 25,0% vs. 57,9% (p=0,016). Снижение ПТГ на 50% зафиксировано у 47,4% всей группы; у 42,9% пациентов с исходным ПТГ<600 пг/мл, у 59,1% пациентов с исходным ПТГ 600–1000 пг/мл и только у 23,6% пациентов с более выраженным ВГПТ (p<0,001).

**ЗАКЛЮЧЕНИЕ:**

ЗАКЛЮЧЕНИЕ. Применение этелкальцетида приводило к снижению уровня ПТГ у большинства пациентов на гемодиализе. При умеренном ВГПТ (ПТГ 600–1000 пг/мл) достичь целевого уровня ПТГ можно у большинства пациентов, но при более высоком исходном уровне ПТГ результат снижается. Признаки автономии околощитовидных желез связаны с большим риском недостаточного эффекта этелкальцетида, чем исходный уровень ПТГ.

## Abstract (en)

**BACKGROUND:**

BACKGROUND: The prevalence of SHPT depends on control strategy and target level of PTH. There is insufficient data for a justified choice of goals and strategy for the SHPT correction.

**AIM:**

AIM: A retrospective, multicenter, cohort study was conducted to assess the ethelcalcetide efficacy after six months in real world practice. The study sought to identify whether MBD-CKD characteristics are predictive of achieving the goals.

**MATERIALS AND METHODS:**

MATERIALS AND METHODS: A study included 302 patients in 20 dialysis units in St. Petersburg. The primary endpoint was the proportion of patients who reached the target of PTH (300–599 pg/ml). The secondary endpoint was an absolute and relative decrease in PTH. The results were compared in subgroups with PTH (<600, 600–1000, ≥1000 pg/ml) and with and without signs of parathyroid authonomy.

**RESULTS:**

RESULTS: In patients aged 56 (12) years (men — 61%) with a dialysis duration of 36 (Q1-Q3 23-55) months with baseline PTH level of 729 (548–957) pg/ml and with calcemia and phosphatemia 2.35 (0.25) and 1.97 (0.47) mM/L, the proportion of success was 49.7%. It is more expected for the PTH of 600–1000 pg/ml compared with PTH>1000 pg/ml: 79% vs. 36%, p<0.001. In presence of signs of parathyroid authonomy, 48% reached the target range vs. 86% in its absence (p<0.001) in subgroup with PTH of 600–1000 pg/ml. For patients with PTH above 1000 pg/ml, the proportion was 25% vs. 58% (p=0.016). The signs of parathyroid authonomy was the most significant independent risk for target achieving (OR=0.3 [95% CI 0.13; 0.68], p=0.004)

**CONCLUSION:**

CONCLUSION: When transferring patients from cinacalcet to etelcalcetide, no new side effects or drug intolerance were noted. In moderate SHPT (PTH 600–1000 pg/ml) it is possible to achieve the target in most patients, but with higher level, the result worsened. The signs of parathyroid authonomy predicts the insufficient effect better than high baseline PTH level.

## ОБОСНОВАНИЕ

Вторичный гиперпаратиреоз (ВГПТ) развивается на ранних стадиях хронической болезни почек (ХБП), и его распространенность постепенно увеличивается по мере прогрессирования заболевания, конкурируя только с низкообменными остеодистрофиями [1] — в зависимости от избранной тактики контроля гиперпаратиреоза, в первую очередь — целевого уровня ПТГ [2]. Плохо контролируемый ВГПТ увеличивает риск переломов, сердечно-сосудистых заболеваний и смерти [3]. В то же время избыточное подавление функции околощитовидных желез способно привести к низкообменным остеодистрофиям, тяжелыми последствиями которых является сосудистая кальцификация и переломы [4]. Коррекция гиперфосфатемии, поддержание нормального уровня кальция и уровня паратгормона (ПТГ) в сыворотке крови в диапазоне величин, в два–девять раз превышающих верхнюю границу нормы, являются рекомендуемой стратегией ведения пациентов с ВГПТ. Медикаментозной составляющей этой стратегии является применение кальцитриола/синтетических аналогов витамина D или кальцимиметиков [5].

В метаанализе 27 рандомизированных контролируемых испытаний (РКИ), кальцимиметики снижали уровень ПТГ (средневзвешенная разность на 178 пг/мл, 95%ДИ: 239–118 пг/мл, p<0,00001), кальция (на 0,18 ммоль/л, 0,22–0,14 ммоль/л, Р<0,00001), уровень фосфатов (на 0,10 ммоль/л, 0,18–0,03 ммоль/л, p=0,008), произведения концентраций кальция и фосфатов (на 0,62 ммоль²/л², 0,78–0,47, p<0,00001). Кальцимиметики повышали уровень костной щелочной фосфатазы и долю пациентов, достигших целевого уровня ПТГ, а также снижали уровень остеокальцина и частоту паратиреоидэктомии. С другой стороны, кальцимиметики в сравнении с плацебо демонстрировали большую общую частоту нежелательных явлений, в первую очередь, гипокальциемии и побочных эффектов со стороны желудочно-кишечного тракта (тошнота, рвота, боли в животе и диарея). Частота серьезных нежелательных явлений между группой, принимавшей кальцимиметики, и контрольной группой не различалась [6].

Прямых сравнений между разными классами препаратов, влияющих на параметры минеральных и костных нарушений при ХБП (МКН-ХБП), проведено немного, поэтому Liu X et al. (2024) использовали аппарат сетевого метаанализа, в который было включено 21 РКИ с участием 4653 пациентов. Не было обнаружено статистически значимой разницы между двумя активными аналогами витамина D и между тремя кальцимиметиками в контроле уровней ПТГ и фосфатов. По сравнению с плацебо кальцитриол и парикальцитол повышали кальциемию, а цинакальцет и этелкальцетид — снижали — даже при совместном применении с активными аналогами витамина D. Эвокальцет снижал содержание кальция и фосфатов в большей степени, чем цинакальцет. По сравнению с парикальцитолом цинакальцет значительно повышает уровень ЩФ, в том числе ее костной фракции. Частота желудочно-кишечных расстройств у цинакальцета и этелкальцетида была значительно выше, чем у парикальцитола. Этелкальцетид и эвокальцет вызывали меньшую частоту желудочно-кишечных расстройств, чем цинакальцет [7].

Клиническая практика в реальных условиях весьма разнообразна и отчасти зависит от доступности антипаратиреоидных препаратов и паратиреоидэктомии в регионе [8][9][10]. По материалам Согласительной конференции KDIGO по МКН-ХБП для строго обоснованного выбора целей и тактики коррекции ВГПТ в настоящее время данных недостаточно [5].

В 2018 г. в Санкт-Петербурге в системе Дополнительного лекарственного обеспечения (ДЛО) стало доступно (бесплатно для пациентов) ограниченное количество этелкальцетида, и большинство гемодиализных пациентов, получавших ранее цинакальцет, были переведены на терапию этелкальцетидом. Кроме того, при новых назначениях кальцимиметиков у пациентов на гемодиализе (ГД) использовался этелкальцетид (пациенты на перитонеальном диализе продолжали лечение или получали новые назначения цинакальцета).

## ЦЕЛЬ ИССЛЕДОВАНИЯ

Настоящее ретроспективное когортное многоцентровое исследование было проведено для оценки результатов применения этелкальцетида в условиях реальной клинической практики. Гипотезой исследования стало предположение о том, что этелкальцетид будет эффективно снижать уровень ПТГ через полгода после начала терапии у пациентов с ХБП С5 на гемодиализе, имеющих ВГПТ.

## ПАЦИЕНТЫ И МЕТОДЫ

## Место и время проведения исследования

В ретроспективное когортное исследование были включены пациенты на гемодиализе, получавшие этелкальцетид в 2018–2019 гг. в 20 отделениях диализа г. Санкт-Петербурга. Критериями включения служили факт обоснованного назначения препарата врачом-нефрологом, а также факт достоверного получения его пациентом в аптечной сети. В исследование вошли 302 пациента, что составило 16% от их общего числа (N=1890), из них 63 пациента (21%) ранее не получали кальцимиметики.

## Дизайн исследования

Исследование многоцентровое, наблюдательное, динамическое, одновыборочное.

Первичной конечной точкой являлась доля пациентов, достигших целевого диапазона ПТГ через 6 мес после начала лечения, который был определен нами как 300–599 пг/мл.

Вторичной конечной точкой исследования являлось снижение ПТГ к концу периода наблюдения в абсолютных и относительных (% снижения от исходного уровня) значениях. В анализе по подгруппам сравнивали частоты достижения первичной и вторичной конечных точек в подгруппах с разным исходным уровнем ПТГ и с признаками автономной аденомы околощитовидных желез (ОЩЖ) и без них.

Основой для составления базы данных послужила систематизированная информация о назначении терапии этелкальцетидом, полученная из регистра Городского нефрологического центра Санкт-Петербурга, дополненная и уточненная информацией из центров диализа. Результаты рутинного наблюдения в диализных центрах были источником информации об уровнях кальция (Са) и неорганических фосфатов (Р) в крови (ежемесячно), паратгормона (ПТГ) — ежеквартально или чаще в период коррекции терапии.

УЗИ ОЩЖ проводилось в начале лечения, перед переводом с цинакальцета на этелкальцетид. Пациенты, начавшие терапию этелкальцетидом, были разделены на 3 группы на основании уровня ПТГ: <600 пг/мл, 600–1000 пг/мл и >1000 пг/мл, а также на основании данных ультразвукового исследования: без достоверных признаков автономии ОЩЖ и с таковыми признаками (объем железы более 500 мм³ или максимальный линейный размер более 10 мм в сочетании с качественной оценкой кровотока по железе, подтверждавшей предположение об автономном росте).

Особенности реальной практики назначения этелкальцетида определялись его ограниченным количеством (в расчете на год), доступного по системе Дополнительного лекарственного обеспечения. По организационным причинам у большинства пациентов, ранее принимавших цинакальцет, перед началом терапии этелкальцетидом имел место перерыв в 1–2 мес (незапланированный период отмывки).

## Статистический анализ

Размер выборки для исследования был ограничен объемом фактически доступных данных, расчет размера выборки не проводился. Для оценки распределения количественных признаков визуально анализировали частотные диаграммы и квантильные графики. При отсутствии выраженных отклонений от нормального распределения описывали данные при помощи среднего и стандартного отклонения: М (SD). В иных случаях приводили медиану, границы первого и третьего квартилей, Me (Q1–Q3), минимум и максимум.

Для оценки динамики уровня ПТГ применяли смешанные линейные модели со случайным эффектом «субъект». Апостериорные сравнения проводили при помощи линейных контрастов с поправкой Холма. Оценку связи исходных величин демографических параметров и параметров минеральных и костных нарушений с конечными точками исследования проводили при помощи многофакторной логистической регрессии. Для оценки соблюдения допущений регрессионного анализа использовали графики остатков и фактор инфляции дисперсии. В соответствии с рекомендациями SAMPL, как метрику качества подгонки (goodness of fit) регрессионных моделей указывали R².

Связь между категориальными факторами оценивали с помощью точного критерия Фишера, теста Фишера-Фримена-Гальтона и теста маргинальной однородности Стюарта-Максвелла (при оценке признаков в динамике).

Анализ проведен в R 4.4.2 и SPSS 21. Результаты считали значимыми при p<0,05.

## Этическая экспертиза

Комитетом по биомедицинской этике Клиники высоких медицинских технологий им. Н.И. Пирогова СПбГУ дано заключение о том, что в связи с ретроспективным характером исследования оно не нуждается в этической экспертизе (выписка из протокола № 06/24 от 20.06.2024 г).

## РЕЗУЛЬТАТЫ

Характеристика группы пациентов, начавших получать этелкальцетид, представлена в таблице 1.

**Table:** Таблица 1. Характеристика группы пациентов, начавших получать этелкальцетид (N=302)

	Пациенты, начавшие получать этелкальцетид, n=302
Основной диагноз	
сосудистые нефропатии	16 (5,3%)
поликистоз почек	35 (11,6%)
сахарный диабет	12 (4,0%)
системные болезни	31 (10,3%)
хронический гломерулонефрит	144 (47,7%)
интерстициальные болезни и мочекаменная болезнь	64 (21,2%)
возраст, лет	56(12)
мужчины/женщины, (%)	184 (61%) / 118 (39%)
срок диализа, месяцев	36 (23–55), от 6 до 132
исходная кальциемия, ммоль/л	2,35 (0,25)
исходная фосфатемия, ммоль/л	1,97 (0,47)
исходный ПТГ, пг/мл	729 (548–957), от 287 до 1473

Во всех трех группах, соответствующих избранным диапазонам исходного ПТГ, мы отметили выраженное его снижение (рис. 1). Один пациент исходно имел ПТГ 287 пг/мл и не был включен в этот этап анализа. Разность средних между этапами «до лечения» и «после лечения» составила для группы с исходным уровнем 300≤ПТГ<600 пг/мл — 222 пг/мл [ 95% ДИ 202; 243], p<0,001; для 600≤ПТГ<1000 пг/мл — 398 пг/мл [ 95% ДИ 373,0; 422], p<0,001; для 1000≤ПТГ пг/мл — 530,8 пг/мл [ 95% ДИ 490; 572], p<0,001.

**Figure:**
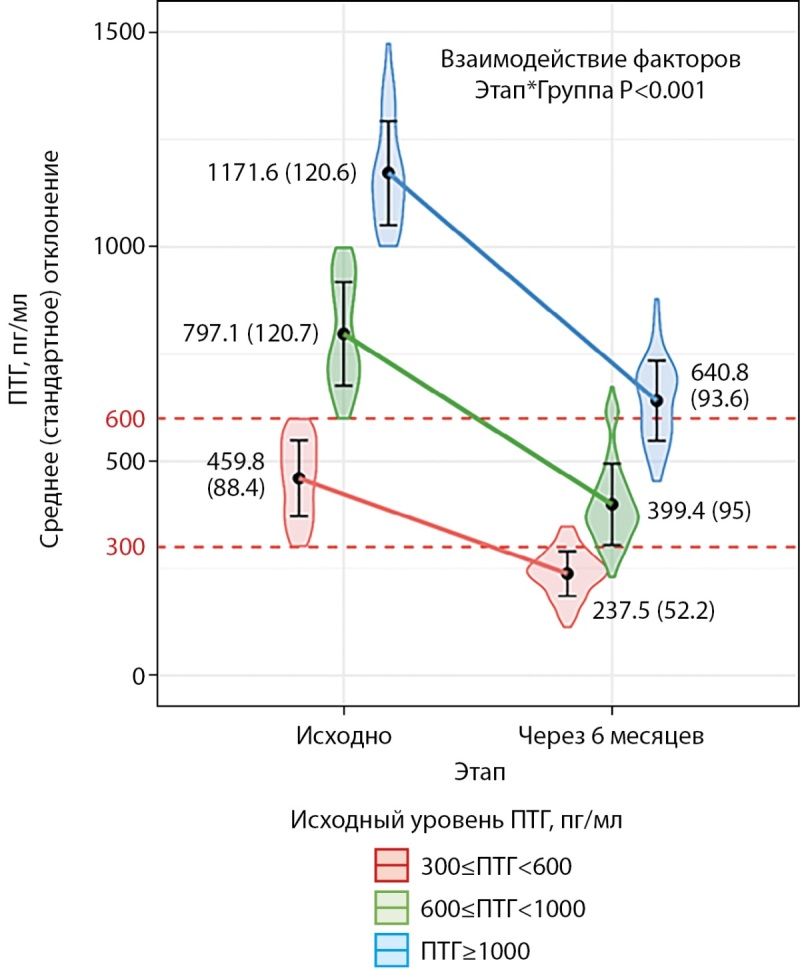
Рисунок 1. Динамика ПТГ в зависимости от исходного уровня. Форма фигур отражает распределение признака. Смешанная линейная модель. R² для фиксированных эффектов (этап и группа) 0,883, для фиксированных и смешанного эффекта (пациент) 0,890. ПТГ — паратгормон. Взаимодействие факторов: Этапы — исходно и через 6 месяцев, Группы — по исходному уровню паратгормона <600, 600≤ПТГ<1000 пг/мл, ≥1000 пг/мл.

Различия в динамике ПТГ были статистически значимы: чем выше был исходный ПТГ, тем более выраженно он снижался.

В отличие от динамики абсолютных значений ПТГ, представленной на рисунке 1, доля снижения ПТГ в ходе лечения максимальна в средней группе с исходным ПТГ 600–1000 пг/мл, а минимальна — в группе с исходным ПТГ ≥1000 пг/мл (табл. 2).

**Table:** Таблица 2. Относительное снижение паратгормона на фоне терапии этелкальцетидом в разных подгруппах пациентов

Группа	N	% снижения, M (SD)	p в дисперсионном анализе
Вся группа	302	48,7 (6,4)%	
Подгруппы по уровню исходного паратгормона
(1) Исходный ПТГ <600 пг/мл	97	48,4 (5,8)%	<0,001
(2) Исходный ПТГ от 600 и менее 1000 пг/мл	150	50,3 (5,6)%¹
(3) Исходный ПТГ ≥1000 пг/мл	55	48,4 (5,8)%², ³
Различия между группами при попарном сравнении	¹отличие от группы (1) p=0,037²отличие от группы (1) p=0,002³отличие от группы (2) p<0,001
Подгруппы по наличию признаков автономии околощитовидных желез
Признаков не найдено	239	49,8 (6,0)%	<0,001
Признаки выявлены	63	44,7 (6,4)%
Попарные сравнения проведены с поправкой Холма

Снижение ПТГ на 50% зафиксировано у 47,4% всей группы; у 42,9% пациентов с исходным ПТГ<600 пг/мл, у 59,1% пациентов с исходным ПТГ от 600 и менее 1000 пг/мл и только у 23,6% пациентов с более выраженным ВГПТ на старте исследования (p<0,001).

Из 302 пациентов 63 (21%) имели признаки автономии ОЩЖ. Это потенциально могло влиять на результаты терапии (динамику ПТГ). В смешанной линейной регрессионной модели трехфакторное взаимодействие «[этап]*[группа по исходному ПТГ]*[признаки автономии]» было статистически незначимо: p=0,961. Для фиксированных эффектов (этап, группа, автономия) R² составил 0,898, для фиксированных и смешанного эффекта (пациент) 0,902. В регрессионной модели без учета исходного уровня ПТГ взаимодействие [этап]*[признаки автономии] было статистически значимым: p<0,001 (рис. 2). Иными словами, при наличии признаков автономии ОЩЖ можно ожидать более выраженное абсолютное снижение ПТГ, чем при их отсутствии. При этом учет категории по исходному уровню ПТГ не меняет эту зависимость. При отсутствии автономии ОЩЖ разность средних для уровня ПТГ составила 338 пг/мл [ 95% ДИ 305; 371], p<0,001, при наличии автономии — 469 пг/мл [ 95%ДИ 408; 529], p<0,001. Таким образом, ожидаемое различие в динамике составило 131 пг/мл [ 95% ДИ 59; 202], p<0,001.

**Figure:**
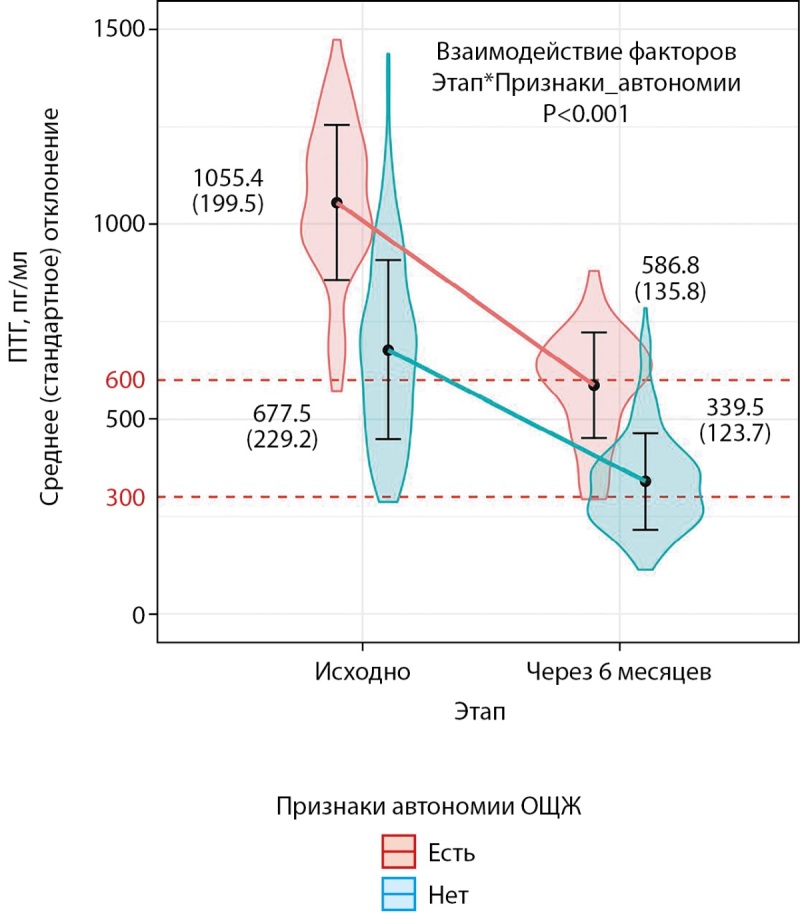
Рисунок 2. Динамика ПТГ в зависимости от наличия признаков автономии околощитовидной железы. Смешанная линейная модель со случайным эффектом «субъект». Форма фигур отражает распределение признака. R² для фиксированных эффектов (этап, аденома) 0,604, для фиксированных и смешанного эффекта (пациент) 0,604. ПТГ — паратгормон; ОЩЖ — околощитовидные железы. Взаимодействие факторов: этапы — исходно и через 6 месяцев; признаки автономии ОЩЖ (нет — есть).

Несмотря на более выраженное снижение абсолютных значений ПТГ при наличии признаков автономии ОЩЖ, такие пациенты реже достигали целевого диапазона значений ПТГ: в группе с исходным уровнем паратгормона 600–1000 пг/мл доля таких пациентов составила 48,0% vs. 85,6% в отсутствии признаков автономии ОЩЖ (p<0,001). Соответствующее соотношение для пациентов с исходным ПТГ выше 1000 пг/мл составляет 25,0% vs. 57,9% (p=0,016 (табл. 3).

**Table:** Таблица 3. Достижение целевого уровня паратгормона в зависимости от наличия признаков автономии околощитовидной железы в группах с разным исходным уровнем ПТГ

Исходный уровень паратгормона	N	Достижение целевого диапазона (300–599 пг/мл)	Все	Признаки автономии околощитовидной железы	p-value
нет	есть
менее 600 пг/мл	97	нет	86	86 (100%)	0 (0%)	0,012
да	11	9 (82%)	2 (18%)
от 600 и менее 1000 пг/мл	150	нет	31	18 (58%)	13 (42%)	<0,001
да	119	107 (90%)	12 (10%)
≥1000 пг/мл	55	нет	35	8 (23%)	27 (77%)	0,016
да	20	11 (55%)	9 (45%)
≥600 пг/мл	205	нет	66	26 (39%)	40 (61%)	<0,001
да	139	118 (85%)	21 (15%)
Все	302	нет	152	112 (74%)	40 (26%)	<0,001
да	150	127 (85%)	23 (15%)

Также при наличии признаков автономии ОЩЖ уровень паратгормона снижался за период исследования от более высоких значений на меньшую долю: процент снижения ПТГ составил 45% (40–50%) против 50% (45–54%) соответственно (табл. 2). Количество пациентов, у которых был достигнут целевой уровень ПТГ при исходном уровне выше 600 пг/мл, также было разным (81,9% в группе без признаков автономии против 34,4% в группе с такими признаками, p<0,001).

Не было выявлено существенной разницы в эффекте терапии в зависимости от количества выявленных ОЩЖ.

На фоне терапии мы не отметили переходов в группу с более высоким уровнем ПТГ: все пациенты или остались в исходной категории, или перешли в категорию более низкого уровня ПТГ. При этом изменение категории было статистически значимо более ожидаемо, чем ее сохранение (тест Стюарта-Максвелла, p<0,001) (рис. 3).

**Figure:**
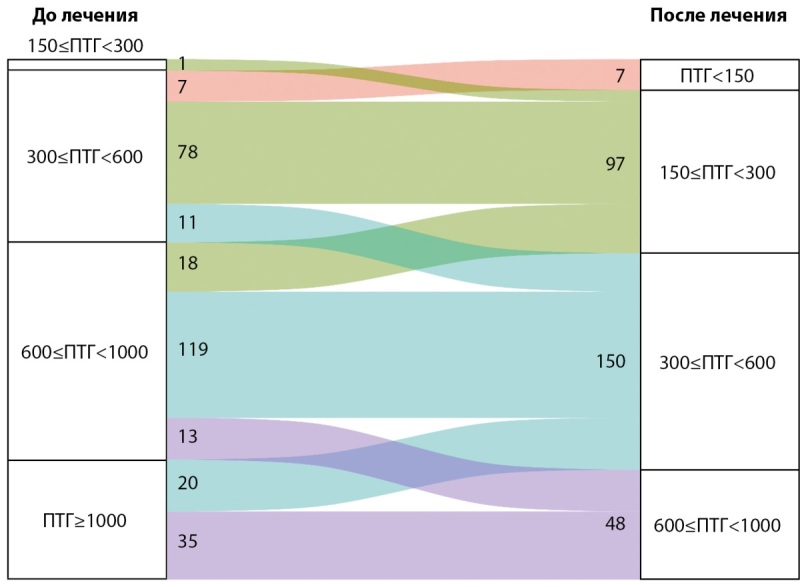
Рисунок 3. Переходы пациентов по категориям паратгормона на фоне терапии этелкальцетидом (N=302). ПТГ — паратгормон, пг/мл.

В целом, 150 пациентов (49,7%) к концу периода наблюдения (6 месяцев) находились в целевом диапазоне ПТГ.

Частота достижения целевого уровня ПТГ (300≤ПТГ<600 пг/мл) более ожидаема для исходной категории 600≤ПТГ<1000 пг/мл по сравнению с категорией ПТГ≥1000 пг/мл: 119/150 (79,3%) против 20/55 (36,4%) соответственно, p<0,001.

В сравнении не учтена группа пациентов с исходным уровнем ПТГ ниже 600 пг/мл, где уровни ПТГ у части пациентов исходно находились в избранном для анализа целевом диапазоне. Среди пациентов этой группы только 11 / 97 (11,3%) оставались в целевом диапазоне, а 78 / 97 (80,4%) пациентов вышли из него в потенциально связанную с рисками развития низкообменных остеодистрофий зону уровня ПТГ 150–300 пг/мл. У семи пациентов ПТГ снизился в диапазон ниже 150 пг/мл.

Переходы пациентов между категориями в зависимости от наличия или отсутствия признаков автономии околощитовидных желез представлены в таблице 4.

**Table:** Таблица 4. Изменение числа пациентов в подгруппах с разным уровнем паратгормона в зависимости от наличия признаков автономии околощитовидных желез за 6 месяцев терапии этелкальцетидом

Категория ПТГ (пг/мл)	Все пациенты (N=302)	Признаков автономии нет(N=239)	Признаки автономии есть(N=63)
до	после	до	после	до	после
<150	0	7	0	7	0	0
150≥ПТГ<300	1	97	1	95	0	2
300≥ПТГ<600	96	150	94	127	2	23
≥600	205	48	144	10	61	38

Анализ вероятности достижения целевого диапазона ПТГ проводили в отношении пациентов с исходным уровнем ПТГ 600 пг/мл и более (205 пациентов), из которых 150 пациентов начали терапию при ПТГ 600–1000 пг/мл, а 55 пациентов — при ПТГ>1000 пг/мл.

В многофакторной логистической регрессии продемонстрирована связь шансов на достижение целевого диапазона ПТГ в ходе лечения, включающего этелкальцетид (табл. 5).

**Table:** Таблица 5. Шансы на достижение целевого диапазона паратгормона, многофакторная логистическая регрессия. R² 0,387

Параметр	ОШ	95% ДИ	P value
Группа паратгормона >1000 пг/мл vs. 600-1000 пг/мл	0,41	0,17–0,99	0,045
Наличие признаков автономии по УЗИ	0,30	0,13–0,68	0,004
Возраст, +1 год	0,99	0,96–1,01	0,313
Ln (Длительность диализа, месяцы ) +1	0,62	0,33–1,13	0,124
Кальциемия, +0,1 ммоль/л	0,86	0,73–1,00	0,059
Фосфатемия, +0,1 ммоль/л	0,92	0,85–1,00	0,046

Наличие признаков автономии ОЩЖ является наиболее значимым независимым предиктором достижения целевого уровня ПТГ: наличие УЗИ-признаков автономного роста узлов околощитовидной железы снижает эти шансы на 70%. Исходный уровень ПТГ выше 1000 пг/мл значимо связан с меньшими на 59% шансами на достижение целевого диапазона ПТГ, по сравнению с исходным ПТГ от 600 до 1000 пг/мл.

На основе модели, представленной в таблице 5, мы создали интерактивное приложение с веб-интерфейсом [11], которое моделирует вероятность достижения целевого диапазона ПТГ. Помимо этого, данное приложение позволяет оценить связь каждого из представленных в таблице факторов при заданных значениях остальных факторов.

Процент пациентов с легкой гипокальциемией (1,9≤Са<2,1 ммоль/л) во время лечения этелкальцетидом составил 18,3%, а с тяжелой гипокальциемией (<1,9 ммоль/л) — 5,8%. Кроме того, при увеличении дозы этелкальцетида и сопутствующей коррекции дозы витамина D распространенность легкой гипокальциемии оставалась практически неизменной (17,4%, р>0,1), в то время как распространенность тяжелой гипокальциемии значительно снизилась (1,9%, p=0,04).

Медиана недельной дозы этелкальцетида составляла 15 (7,5–15) мг (диапазон доз 7,5–22,5 мг) и не различалась у пациентов, не принимавших ранее цинакальцет или перешедших с него после вынужденного периода отмывки.

После перевода пациентов с цинакальцета на этелкальцетид ни у одного из них не развилась клиническая непереносимость или новые побочные эффекты. Коэффициент пересчета дозы для перехода составил [этелкальцетид в неделю] = 0,277×[мг цинакальцета в день], что указывает на то, что разовая доза этелкальцетида (2,5 мг за сеанс) функционально несколько меньше, чем разовая доза цинакальцета (30 мг в день).

## ОБСУЖДЕНИЕ

Клиническое значение превышения целевых уровней паратгормона для пациентов на заместительной почечной терапии, как и сами целевые уровни, к настоящему времени не определены окончательно. Возможно, единого решения для большинства пациентов и не существует. Например, в недавнем реанализе большой базы данных европейского 3-летнего проспективного наблюдательного исследования COSMOS (6797 пациентов из 227 случайно отобранных диализных центров в 20 европейских странах) установлено, что превышение 9-кратной величины верхней границы нормы было достоверно связано с более высоким относительным риском смерти только у пациентов с сахарным диабетом, но не у пациентов без диабета [ 1,53 (95% ДИ 1,07–2,19) и 1,17 (95% ДИ 0,91–1,52)]. При этом наличие или отсутствие сахарного диабета не влияло на связь относительного риска смерти с уровнем кальция или фосфата в сыворотке крови [12]. В нашей группе диагноз основного заболевания не был связан ни со снижением ПТГ (абсолютным или относительным) на фоне терапии этелкальцетидом, ни с долей пациентов, достигших целевого диапазона ПТГ.

Выбор целевого для данного исследования уровня ПТГ обусловлен рядом аргументов как медицинского, так и организационного характера. В отсутствие прямых контролируемых исследований по преимуществам выбора того или иного целевого диапазона его избирают исходя из результатов post-hoc анализа РКИ и крупных эпидемиологических исследований. Наименьшее значение риска смерти в большинстве из них связывают с уровнем ПТГ около 400 пг/мл [13][14]. В исследовании ARO-II (N=8817) с наименьшими рисками был связан диапазон 239–710 пг/мл. Как дальнейшее снижение ПТГ для пациентов с исходным уровнем паратгормона ниже этого диапазона, так и выход вниз для пациентов, пребывавших в этом диапазоне, был связан был с увеличением рисков. Напротив, рост ПТГ для первой группы пациентов был связан со снижением рисков. Более того, умеренное повышение ПТГ (на 100–200 пг/мл) для пациентов из диапазона 239–710 пг/мл также был связано с небольшим, но значимым снижением рисков [14]. В России, странах Ближнего Востока, Канаде и США, северных странах Европы доля пациентов с ПТГ в диапазоне 300–600 пг/мл превышает таковую с ПТГ 150–300 пг/мл [15]. Из-за объективных ограничений в Санкт-Петербурге сложилась практика относительно редкого контроля паратгормона (как правило, не чаще 1 раза в квартал, в том числе, в период коррекции доз антипаратиреоидных препаратов), что не отличается, впрочем, от распространенной практики в мире [16]. Это обстоятельство, а также низкая доступность ультразвукового исследования околощитовидных желез (и сцинтиграфии) в отсутствие твердых свидетельств преимуществ антипаратиреоидной терапии при умеренном ВГПТ (300–600 пг/мл) обусловила выбор нижней границы целевого диапазона для активной антипаратиреоидной фармакотерапии в 300 пг/мл.

Верхняя условная граница для начала терапии была принята в 1500 пг/мл в качестве уровня, с высокой вероятностью свидетельствующего о наличии автономии ОЩЖ, мало чувствительной к любой терапии. Даже снижение ПТГ на 30–40% (типичное для терапии в средних дозах [6][17][18]) не приводит пациента к целевому диапазону, и потребность в инвазивных вмешательствах сохраняется [8][19]. Верхний уровень целевого диапазона, которого следует достичь в ходе терапии, определен в 600 пг/мл — как наиболее часто выявляемый предел, далее которого следует увеличение рисков неблагоприятных исходов. Кроме того, именно его чаще всего используют в исследованиях реальной клинической практики по этелкальцетиду последних лет; это обеспечивает сравнимость результатов [8].

Именно неизбирательное применение первого кальцимиметика цинакальцета привело, вероятно, к нейтральным результатам в крупнейшем исследовании EVOLVE [20]. Только в отдельных подгруппах и при определенных условиях в post hoc анализе были получены свидетельства преимуществ цинакальцета: улучшение (или тенденция к улучшению) сердечно-сосудистых исходов получено: (1) при ограничении времени наблюдения полугодом после приема последней дозы препарата; (2) у пациентов старше 65 лет; (3) при снижении в ходе терапии уровня FGF23; (4) при учете только «не-атеросклеротических» сердечно-сосудистых событий; (5) у пациентов с исходным ПТГ ниже 900 пг/мл; (6) при исходном сроке диализа менее 2 лет [20]. Спустя годы после публикации EVOLVE авторы аналитических обзоров вынуждены констатировать: несмотря на многообещающие перспективы кальцимиметиков в отношении замедления прогрессирования сердечно-сосудистой патологии, улучшения остеогенеза, уменьшения количества кальципротеиновых частиц в сыворотке крови (участвующих в прогрессировании эндотелиальной дисфункции, атерогенеза и кальцификации сосудов), «однозначных доказательств нет» [21].

При этом кальциемия ниже 2,1 ммоль/л в крупном (23 тысячи пациентов) и продолжительном (медиана 108 месяцев) исследовании оказалась связана с риском смерти — несколько большим при терапии цинакальцетом (+32%), чем без нее (+15%) [22]. А более высокие дозы кальцимиметиков связаны с большей частотой гипокальциемии [19][21][23][24].

В регистрационных РКИ, опубликованных при введении этелкальцетида в клиническую практику, представлено лишь сравнение с плацебо и с цинакальцетом в отношении суррогатных исходов: достижение целевых показателей МКН-ХБП [17][18][20][21]. Первичной конечной точкой в этих исследованиях была доля пациентов со снижением исходного уровня ПТГ на 30% и более. Она достигнута у 72% (vs. 62% для цинакальцета) при исходном ПТГ<900 пг/мл и у 64% (vs. 53%) при исходном ПТГ≥900 пг/мл. Интересно отметить, что при терапии этелкальцетидом ровно на такую же величину чаще (на 10%) встречалась гипокальциемия самый распространенный побочный эффект. Это наводит на мысль о том, что различия в эффективности связаны были с приверженностью терапии (внутривенное введение vs. пероральной терапии). Полученные в этих РКИ доли достижения цели были несколько выше, чем в нашем исследовании, но наши результаты были очень близки недавнему российскому рандомизированному исследованию по сравнению этелкальцетида с цинакальцетом — снижение на 55,4% vs. 50,2% [25].

Помимо подавления активности ОЩЖ, этелкальцетид эффективен также в снижении сывороточных уровней кальция, фосфатов и фактора роста фибробластов 23 (FGF23). За 8 лет присутствия препарата на рынке до настоящего времени лишь в нескольких исследованиях сообщалось о клинически значимых исходах. Предварительные данные свидетельствуют о его роли в снижении скорости костного обмена, повышении минеральной плотности костной ткани и сохранении структуры кости, что указывает на возможное положительное влияние на почечную остеодистрофию [26]. Получены данные за то, что терапия этелкальцетидом связана со значительным замедлением прогрессирования гипертрофии левого желудочка (правда, только в сравнении с терапией альфакальцидолом, но не плацебо) [27]. Помимо снижения FGF23, этелкальцетид повышает уровень склеростина в сыворотке крови. Эти данные свидетельствуют о возможном благоприятном влиянии на сердечно-сосудистую систему [28]. Но исследования, специально предназначенные для оценки его роли в снижении числа переломов, сердечно-сосудистых заболеваний и смертности от всех причин по-прежнему отсутствуют [29].

Возможно, одной из причин такого дефицита исследований является сохраняющаяся неопределенность в границах целевого диапазона. После перехода от цинакальцета к этелкальцетиду (и другим кальцимиметикам) анализ последующей реальной клинической практики применения этелкальцетида с сравнении с цинакальцетом продемонстрировал достижение целевого уровня ПТГ (<600 пг/мл) у 86% пациентов при исходном ПТГ<800 пг/мл), у 59% (при ПТГ 800–1000 пг/мл) и у 58% (при ПТГ>1000 пг/мл). Соответствующие доли для начавших терапию цинакальцетом составили 62%, 64% и 41%. Среди 186 пациентов, переведенных с цинакальцета на этелкальцетид доля достигающих целевого уровня ПТГ увеличилась с 22% в месяц перед переводом до 51% через 6 месяцев [30]. Встречаются и исследования с более скромными результатами применения этелкальцетида на фоне более выраженного ВГПТ на старте. Среди 148 пациентов в Сингапуре к 8 месяцам терапии целевого уровня в <600 пг/мл достигли только 26% пациентов, а снижение ПТГ к 4 месяцам составило 17% [8]. Еще 29% находились в диапазоне 600–1000 пг/мл, а почти половина — выше него. Достижимость цели оказалась связанной с национальностью пациентов (китайцы/индусы/малайцы). Наибольшая часть пациентов (66%) стартовала с дозы 7,5 мг/нед (медиана и Q1Q3); M(SD): 9,0(3,8 мг); максимальные за исследование дозы составили 7,5 и 15 мг/нед (по 39%), медиана 15 (7,5, 15), что связано было с ограничениями по возмещению расходов на этелкальцетид.

В работе Karaboyas A et al. [31] средний исходный уровень ПТГ составлял 671 (580 пг/мл, их группа скорее соответствовала нашим первым двум подгруппам (ПТГ<1000 пг/мл) как по исходным величинам ПТГ, так и по результату вмешательства. Следует отметить, что по нашим данным с результатами терапии этелкальцетидом выраженно связан не только исходный уровень ПТГ, но и наличие признаков автономии околощитовидных желез: доля достигших целевого диапазона в ходе терапии среди пациентов с признаками автономии ОЩЖ существенно ниже, чем у пациентов без них (34,4% против 89,1% соответственно). Кроме того, в полученной нами регрессионной модели наличие признаков автономии ОЩЖ было статистически значимо связано с 70% снижением шансов на достижение целевого диапазона ПТГ, являясь наиболее значимым фактором риска из всех включенных в модель. В крупном РКИ с цинакальцетом (EVOLVE) во вторичном анализе преимущества в твердых исходах получили пациенты, которые лечились менее двух лет и исходный уровень ПТГ у которых не превышал 900 пг/мл, то есть, при условиях, значительно снижающих шанс на существование автономной аденомы ОЩЖ. Напрямую этот вывод не сформулирован в публикациях, посвященных EVOLVE, а в отношении этелкальцетида подобной аналитики в доступной литературе мы не нашли.

Доступность препарата может оказывать влияние на тактику его применения и результаты не только в развивающихся [32], но и развитых странах: в США переход от дополнительной оплаты лекарственной терапии к включению всей терапии в единую («пакетную» — bundle) оплату привел к снижению доли пациентов на терапии этелкальцетидом с 12 до 5% и последующему росту уровня ПТГ на 107 (95% ДИ: 80–133) пг/мл и распространенности уровней ПТГ выше 600 пг/мл на 15% — с 28 до 43% [31].

В немногих работах, где представлен коэффициент пересчета дозы цинакальцета и этелкальцетида указываются близкие нашему [мг этелкальцетида в неделю] = 0,277×[мг цинакальцета в день]) значения: [мг этелкальцетида/сеанс] = 0,111*[мг цинакальцета в день] + 0,96, R² = 0,57 [33]. Близость данных различных исследований может облегчить планирование перевода пациентов между разными препаратами из расширяющегося перечня кальцимиметиков.

## Клиническая значимость результатов

В условиях ограниченной доступности дорогостоящего медикаментозного лечения пациентов с ВГПТ при его использовании предпочтение целесообразно отдавать пациентам с умеренным повышением уровня ПТГ (до 1000 пг/мл) и без признаков автономии околощитовидных желез. В других случаях терапия может оказаться малоэффективной (целевой диапазон ПТГ не будет достигнут) или дорогостоящей; более рациональными окажутся инвазивные вмешательства.

## Ограничения исследования

Доза этелкальцетида из-за его ограниченного количества в расчете на год на диализную популяцию Санкт-Петербурга была произвольно ограничена величиной в 7,5 мг три раза в неделю. По той же причине этелкальцетид реже применялся для поддержания исходного уровня паратгормона в диапазоне 150–300 пг/мл и не применялся у пациентов с исходным уровнем паратгормона выше 1500 пг/мл, а также при отсутствии удовлетворительного ответа в части уровня паратгормона на начало/возобновление терапии кальцимиметиками (в течение двух месяцев). Таким образом, описанная практика отличается от протокола регистрационных РКИ [23][24], но, возможно, была ближе к реальной практике в других странах и регионах [31][32].

К ограничениям исследования можно отнести выполнение анализов биохимических показателей крови в разных лабораториях. Кроме того, нам доступны были лишь основные показатели, отражающие нарушения минерального обмена (уровень кальция, фосфора, ПТГ), и недоступны биохимические маркеры костного обмена, результаты денситометрии, а также информация о сопутствующей терапии, способной повлиять на уровни маркеров минеральных нарушений (препараты витамина D, фосфат-биндеры). Недоступна была информация по изменению данных ультразвукового исследования ОЩЖ.

## Направления дальнейших исследований

Выводы о факторах, связанных с эффективностью медикаментозной терапии или инвазивных вмешательств, полученные в ретроспективном анализе, необходимо подтвердить в проспективном сравнительном интервенционном исследовании.

## ЗАКЛЮЧЕНИЕ

Применение этелкальцетида приводило к снижению уровня паратиреоидного гормона у большинства пациентов на гемодиализе, которым препарат неизбирательно назначался для контроля вторичного гиперпаратиреоза. При переводе пациентов с цинакальцета на этелкальцетид не было отмечено новых побочных эффектов или непереносимости препарата. Наши результаты указывают на то, что при умеренном ВГПТ (ПТГ 600–1000 пг/мл) достичь целевого уровня ПТГ с помощью применения этелкальцетида можно у большинства пациентов, но при более высоком исходном уровне ПТГ результат снижается. Признаки предполагаемой автономной аденомы околощитовидных желез могут предсказать недостаточный терапевтический эффект этелкальцетида лучше, чем исходный уровень ПТГ.

## ДОПОЛНИТЕЛЬНАЯ ИНФОРМАЦИЯ

Источники финансирования. Работа выполнена по инициативе авторов без привлечения финансирования.

Конфликт интересов. Авторы декларируют отсутствие явных и потенциальных конфликтов интересов, связанных с содержанием настоящей статьи

Участие авторов. Все авторы одобрили финальную версию статьи перед публикацией, выразили согласие нести ответственность за все аспекты работы, подразумевающую надлежащее изучение и решение вопросов, связанных с точностью или добросовестностью любой части работы.

Благодарности. Авторы выражают благодарность Марченко Алене Викторовне за помощь в сборе информации.
